# Crowdsourcing and curation: perspectives from biology and natural language processing

**DOI:** 10.1093/database/baw115

**Published:** 2016-08-08

**Authors:** Lynette Hirschman, Karën Fort, Stéphanie Boué, Nikos Kyrpides, Rezarta Islamaj Doğan, Kevin Bretonnel Cohen

**Affiliations:** ^1^The MITRE Corporation, Bedford, MA, USA; ^2^University of Paris-Sorbonne/STIH Team, Paris, France; ^3^Philip Morris International R&D, Philip Morris Products S.A., Neuchâtel, Switzerland; ^4^Joint Genome Institute, Walnut, Creek, CA, USA; ^5^National Center for Biotechnology Information, National Library of Medicine, National Institutes of Health, Bethesda, MD, USA; ^6^University of Colorado, School of Medicine, Denver, CO, USA

## Abstract

Crowdsourcing is increasingly utilized for performing tasks in both natural language processing and biocuration. Although there have been many applications of crowdsourcing in these fields, there have been fewer high-level discussions of the methodology and its applicability to biocuration. This paper explores crowdsourcing for biocuration through several case studies that highlight different ways of leveraging ‘the crowd’; these raise issues about the kind(s) of expertise needed, the motivations of participants, and questions related to feasibility, cost and quality. The paper is an outgrowth of a panel session held at BioCreative V (Seville, September 9–11, 2015). The session consisted of four short talks, followed by a discussion. In their talks, the panelists explored the role of expertise and the potential to improve crowd performance by training; the challenge of decomposing tasks to make them amenable to crowdsourcing; and the capture of biological data and metadata through community editing.

**Database URL:** http://www.mitre.org/publications/technical-papers/crowdsourcing-and-curation-perspectives

## Introduction

Crowdsourcing, a natural evolution of Web technologies, is attracting increased attention in the biocuration and natural language processing communities as a cost-effective way to develop resources for systems evaluation and machine-learning, to perform specific tasks in biocuration and to collect improved data and metadata (1).

Although the use of crowdsourcing technologies is now widespread, especially in natural language processing, the broader discussion of the applicability to biocuration is just now becoming a central topic within the biocuration community. The Pacific Symposium on Biocomputing 2015 held a session entitled ‘Crowdsourcing and Mining Crowd Data’ (2). Presentations on crowdsourcing have steadily increased at the annual Biocuration conferences: there were three talks at Biocuration 2014 (https://biocuration2014.events.oicr.on.ca/); Biocuration 2015 (http://biocuration2015.big.ac.cn/workshop) included a keynote and a workshop on crowd and community annotation; and at Biocuration 2016 (https://www.isb-sib.ch/events/biocuration2016/scientific-program) there were two workshops on community curation and a session on ‘Crowd and Community Curation’ with four talks. The goal of the panel session at BioCreative V was to build on this interest, bringing together the perspectives of the text mining and natural language processing communities with those of curators and bioinformaticians developing curated resources.

This perspective piece reviews four case studies to explore broader questions, including: expertise and the kinds of expertise required for different tasks; crowdsourcing applied to biocuration that uses a micro-tasking approach combining automated entity extraction with human judgments on relationships between those entities (so-called ‘hybrid curation’); the use of crowdsourcing to verify (and refine) existing network models for disease-related pathways derived from literature curation and transcriptomics data; and the challenges of capturing adequate computable metadata for metagenomics and the need for crowdsourced data. This is a multi-disciplinary highly complex space—the goal of this paper is to encourage further research, by exploring possible ways in which crowdsourcing and text mining could be combined to address major challenges for biocuration, in particular, trade-offs in cost, quality, timeliness and ability to recruit people with appropriate expertise for curation tasks.

## Background: types and approaches to crowdsourcing

Khare et al. (2015) describe a range of crowd-based approaches, including labor markets for micro-tasking (such as Amazon Mechanical Turk), collaborative editing (wikis), scientific games and community challenges (1). The ‘crowd’ involved in these applications ranges from scientists participating in community annotation and evaluation activities to citizen scientists to people participating in crowd labor platforms; these participants differ in expertise and motivation (scientific, entertainment, financial); and the crowdsourced applications differ in intended use, from development of training data to improve algorithms, to validation of curated data and to generation of curated data.

These approaches differ along multiple axes:

- Task complexity, with Games With A Purpose (GWAPs) and collaborative editing activities at the high end, and micro-tasking environments, such as Amazon Mechanical Turk at the lower end of complexity.
- Time per task, which is highly correlated with task complexity.
- Expertise required, which is variable, depending on the application purpose;
- Incentives, which may include contributing to a shared scientific endeavor, reputation building, learning new skills and direct compensation.

Research in this area is still in very early stages. The case studies represent probes into this complex space that can demonstrate feasibility, illuminate challenges and suggest new applications for a crowdsourcing approach applied to biocuration.

## The nature of expertise in the context of crowdsourcing

In annotation projects that combine linguistic annotation (e.g. annotation of syntactic structure or coreference relations) and domain-specific ‘semantic’ annotation, particularly of metadata (e.g. whether or not a pathology report states that a tissue sample is pathological), it has long been recognized that the different tasks may require very different types of expertise—in particular, linguistic expertise and subject-matter (or ‘domain’) expertise. This distinction has been formalized in the ‘mixed annotation model’ (http://amberstubbs.net/docs/AmberStubbs_dissertation.pdf). However, a wider analysis of the issue, including crowdsourcing, suggests that the distinction should be more precise and include expertise of the domain of the annotation (which is usually not linguistics as a whole, but, for example, a certain type of syntax), the domain of the corpus (which can be biomedical, football, etc.) and expertise in the annotation task itself (including understanding of the annotation guidelines and tools).

**Figure 1.**
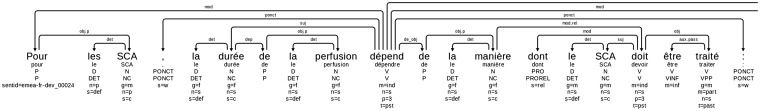
Dependency parse for the sentence ‘For the ACS [Acute Coronary Syndromes], the duration of the IV depends on the way the ACS should be treated: it can last a maximum of 72 h for patients who need to take drugs’ [In the original French: ‘Pour les SCA, la durée de la perfusion dépend de la manière dont le SCA doit être traité: elle peut durer jusqu’à 72 heures au maximum chez les patients devant recevoir des médicaments.’].

The advent of the social Web has made crowdsourcing easier by making it possible to reach millions of potential participants. Various types of Web platforms have been designed, from contributed updates to Wikipedia, to remunerated micro-task applications (3, 4). Crowdsourcing is commonly understood as the act of using a crowd of non-experts (usually via the Web) to perform a task. This assumption raises the question: what exactly is an expert? And more precisely, in our case, what is an annotation expert? To illustrate this, let us take a (real) annotation example from the French Sequoia corpus (5), shown in Figure 1.

In this case, the subcorpus is from the pharmacology domain from the European Medicine Agency, and the annotation is a certain type of linguistics (syntax). Who would be an expert here? A pharmacologist? A linguist? A pharmacist–linguist? Can a native French speaker, without any prior knowledge in syntax or pharmacology, but trained for the task (i.e. a participant in crowdsourcing) be an expert?

This shows that we have to be more precise; we need to distinguish between expertise in the domain of the corpus (pharmacology), expertise in the domain of the annotation (syntax) and expertise in the task (annotating syntactic relations with a certain tool, according to certain guidelines). Being an expert is defined by the on-line Merriam-Webster dictionary as ‘having or showing special skill or knowledge because of what you have been taught or what you have experienced’; our crowdsourcing participant may well correspond to the definition of an expert, as someone (well) trained for the task.

Crowdsourcing annotation is now mature enough to be able to check quality and verify that participants can be experts in the task. Let us take as an example a certain type of crowdsourcing, Games With A Purpose (GWAPs).

GWAPs constitute a very specific crowdsourcing type, in which (i) the participants are not remunerated (unlike microworkers) and (ii) are not necessarily aware of the final product they are creating while playing (unlike Wikipedians or Distributed Proofreaders). It is also important to note that GWAPs readily allow for the training of the participants, whereas this is not necessarily the case for microworking platforms. In microworking platforms, the participants can be tested, and therefore may have motivation to train, but extended training is not planned in the platform as such and workers may perform hidden work, as described in Ref. (6), to get expertise on the task.

GWAPs have proven to be very efficient in producing language data. The first ones were limited to using the players' knowledge of the world, for example, to tag images [ESP game (4)] or to associate ideas [JeuxDeMots (http://jeuxdemots.org/) (7)]. The former enabled the annotation of 350 000 images and the latter generated a lexical network with >46 million relations. Other games were created relying on the players' school knowledge, like Phrase Detectives (https://anawiki.essex.ac.uk/phrasedetectives/) (8), which made it possible to annotate co-references in a 200 000 words corpus, with approximately 84% observed agreement between the players and the reference.

**Figure 2.**
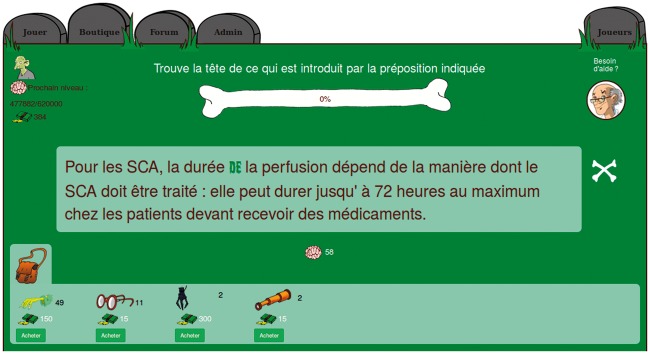
ZombiLingo Interface [Instruction: ‘Find the head of what is introduced by the highlighted preposition’). Sentence: ‘For the ACS [Acute Coronary Syndromes], the duration of the IV depends on the way the ACS should be treated: it can last a maximum of 72 h for patients who need to take drugs’, the right answer is ‘perfusion’ (IV)].

**Figure 3.**
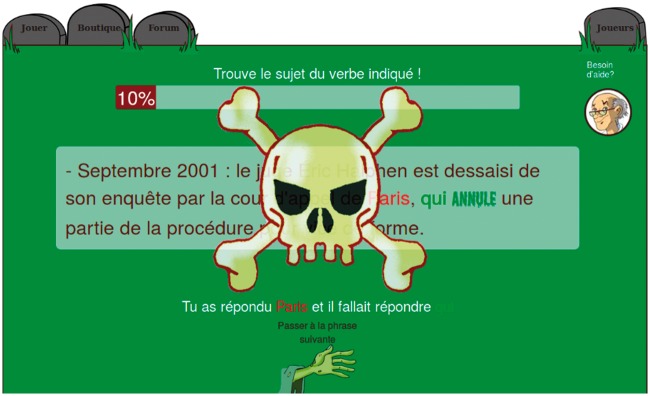
ZombiLingo Training Phase: Correction of a Wrong Answer [Instruction: ‘Find the subject of the highlighted verb’). Correction: **‘**You selected *Paris* while you should have answered *qui (who)’*].

More recently, GWAPs have been developed that address complex tasks which depend on the players' learning capabilities. For example, this is true for FoldIt (http://fold.it) (9), in which it took a team of players (after hours spent on the game to master the task) only a few weeks to find the solution to the crystal structure of a monomeric retroviral protease (simian AIDS-causing monkey virus), an issue unsolved for over a decade. This example and other language-oriented GWAPs inspired ZombiLingo (http://zombilingo.org/) (10), a game in which players, eating ‘heads’, annotate corpora with dependency syntax—see Figures 2 and 3 below, for examples. Despite the fact that the game features in the initial release were still very basic, the game allowed for the production of > 23 000 annotations in one week, with approximately 84% accuracy on average across the 10 more active players (over 86% if removing the best and worst players) (11).

These experiments show that it is possible to use GWAPs to annotate corpora and that these games can produce phenomenal quantities of language data. The quality of this production, when evaluable (i.e. when a reference exists), is remarkably high if the players are well-trained; see Figure 3 below for an example.

A counter-example is that of the annotation of ‘properties’ in Phrase Detectives (12), where the agreement between the players and the reference was close to null. Although at least some players became experts in the anaphora annotation task as presented in Phrase Detectives, none of them managed to master the annotation of properties and achieve expert status on that task. (In the following example, postman is a property of Jon: *Jon, the postman, delivered the letter*.) This is probably due to a lack of training, because the tutorial focused on anaphora rather than properties.

In all cases, relatively few participants produced a lot of language data (12). When the quality of the participants’ data is high, we can consider them to be experts, at least for the task at hand. Crowdsourcing with GWAPs is therefore more about finding experts-to-be from the crowd and training them on the task than using a crowd of non-experts.

Given a well-designed game, with an appropriate training phase and evaluation protocol, there is virtually no end in sight as to what can be done with GWAPs without raising the employment ethical issues involved in microworking crowdsourcing (13); however GWAPs can create other kinds of ethical issues (7, 14) and attracting players remains a real challenge (11).

## Hybrid curation: automated extraction and crowdsourcing

There is an urgent need for an accurate, scalable, cost-effective curation process to overcome the curation bottleneck. This section discusses two experiments that explore what kinds of curation tasks might be amenable to a micro-tasking approach, how to combine human expertise with automated entity-tagging, and what the cost, quality and throughput implications might be. We refer to this approach as ‘hybrid curation’ because it combines text mining for automated extraction of biological entities (e.g. genes, mutations, drugs, diseases) with crowdsourcing to identify relations among the extracted entities (e.g. mutations of a specific gene, or labeling of indications of a specific drug). (There are many ways of combining automated extraction with crowdsourced judgements; we use the term “hybrid curation” here as a short-hand for the two-stage workflow used for these two experiments consisting of automated entity tagging followed by human judgment for relations among entities.) The rationale was to take advantage of what automated information extraction can do well (e.g. entity extraction for multiple types of biological entities), and couple this with micro-tasks that humans can do quickly and well, such as judging whether two entities are in a particular relationship.

**Figure 4.**
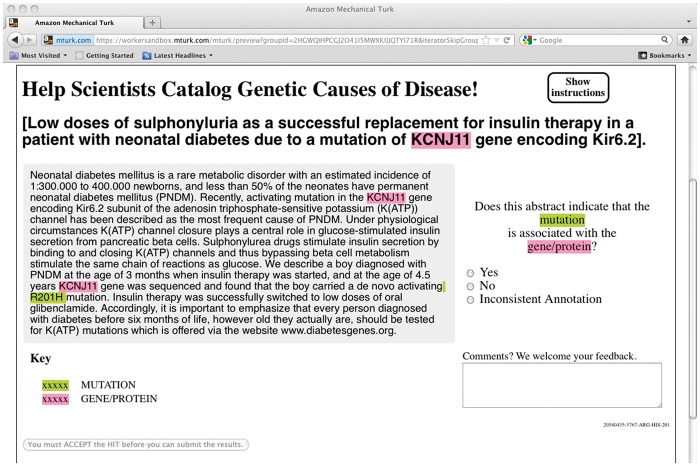
Screenshot of Interface for Judging Gene-Mutation Relations.

The workflow is as follows: the material is prepared by running automated entity extractors over a short text (e.g. an abstract) to produce entity mentions highlighted by type, in their textual context. A pair of entities, with mentions highlighted in the text, is then presented as micro-tasks to a crowd labor platform, where workers are asked to judge whether the highlighted entities are in the desired relationship, as shown in Figure 4. The judgments are then aggregated to provide candidate curated relations that can be deposited into a repository after expert review.

Hybrid curation has many potential applications to biomedical curation problems—for example, to extract gene/mutation associations from the published literature (15), or to extract drug indications (relevant to indexing and search operations over a database such as DailyMed) (http://dailymed.nlm.nih.gov/dailymed/) (16). One of the teams participating in BioCreative V (17) used a hybrid curation approach to extract chemical-induced disease relations for participation in the BioCreative Chemical Disease Relation track. In addition, this approach has been used to create enhanced data sets for machine-learning based systems, with the goal of improving performance of both entity and relation extraction systems in biomedical domains (18, 19).

The attraction of this approach is first, it has the potential to provide cost-effective high throughput curation and second, it greatly simplifies the process of recruiting annotators. The open questions are (1) how to achieve the necessary quality; (2) what kinds of tasks are amenable to this approach; and (3) how to minimize set up costs of the micro-tasks to create a cost-effective, repeatable approach.

The two previously mentioned experiments (15, 16) used the Amazon Mechanical Turk cloud labor platform. Participants (known as ‘Turkers’) were recruited via the crowd labor platform (restricted in these experiments to US participants only); participants were paid 6–7¢ per micro-task. Prior to participating in the task, each Turker had to pass a qualifying exam, consisting of 5–10 sample questions. The exam, in addition to screening out poor performing Turkers, also provided a limited training opportunity. In both experiments, results were evaluated by comparing them with expert-curated gold standard data. In both cases, the tasks included a number of control items (items whose answers were known). Turker judgments were aggregated using a Naïve Bayes approach, based on Turker performance on the control items.

The findings for the gene-mutation extraction experiment (Figure 4) showed that it was possible to achieve reasonable accuracy (recall > 70%, precision ∼80%, at a cost of less than $1 per abstract) by aggregating results from up to 5 Turkers but discarding any Turkers whose performance against control items (items with known answers) was worse than random. These results were promising but the precision was significantly below that achieved by expert curators, reported as ∼90% precision at 70% recall (20, 21).

In the experiment to identify drug indications from drug inserts, the results aggregated across five-fold independent annotation gave good precision (96%) at a recall of 89% and a cost of $1.75 per abstract. Of particular note, the throughput was rapid: the drug labeling experiment took 8 h of elapsed time (from time the task was posted to completion) to obtain five independent judgments for each possible indication in a set of 700 drug inserts.

Conventional wisdom suggests that biocuration tasks require domain expertise; however, the results of both experiments reveal that when the task is structured as carefully designed micro-tasks, it may be possible to leverage the labor of the crowd to achieve rapid throughput and cost-effective curation, especially by adding a layer of expert checking.

These considerations (accuracy, cost and throughput) are the major dimensions in understanding the current curation bottleneck. Manual expert curation is relatively high precision but is not readily scalable and, over the long-term, not affordable as biological databases proliferate and the literature continues to grow exponentially. Manual curation is resource-constrained; it is expensive and time consuming to recruit and train qualified curators and the manual curation process is relatively slow and limited by the availability of curators. For example, for a highly optimized curation workflow it took trained curators about 5.5 min/abstract to curate drug-disease relations. A total of five curators were able to curate a corpus of 89 000 documents over the course of a year. More typical speeds for curation range around 15–20 min/abstract (22). (At $30/curator-hour, this amounted to $2.75–$10/paper – after training and development of supporting infrastructure.) The agreement among expert curators is typically high (90% precision and 70% recall). In contrast, the crowdsourcing approach showed very high throughput and reasonable cost (on the order of $1–3 per abstract); however, task design requires experience and careful planning to develop the right sized micro-tasks, to provide clear instructions and to develop appropriate control items for effective aggregation. Depending on the task, it can achieve results comparable to expert curation, but there are significant start-up costs to set up the pipeline and there are constraints on task complexity, including the amount of context needed for certain decisions. For example, it would be very hard to use this approach to curate long-distance relationships, requiring the display of more text than can easily fit on a single screen. A limitation of the automatic pre-annotation setup is that recall is always dependent on the performance of the automated entity extraction components or taggers. If the selected automatic entity extractor misses an entity (that is, it never gets tagged), then it cannot be shown to Turkers, because Turkers only judge relations between entities tagged by the automated entity extraction systems. If taggers with maximum recall are selected, this will introduce noise, which can drive up cost (i.e. more relations to judge) and even introduce precision errors, if Turkers mistakenly select an incorrectly tagged entity. To ensure quality, effective use of control elements is important—control items sprinkled carefully through the task can help manage the quality of the results by checking that the workers are providing good results. In one simulation experiment, where Turkers were not paid if they performed at < 50% accuracy on control items, the task results became more accurate and the final cost was lower. Finally, it is surprising that, even though the background of crowd workers cannot be selected—therefore, domain expertise is not guaranteed—the aggregated results did show good accuracy, so the issue of defining what kind of expertise is needed for such an annotation task is something that needs to be explored further. This is consistent with earlier work showing that the wisdom of a crowd of non-experts may be comparable to the judgments provided by experts (23).

## Crowdsourcing and higher-level tasks: biological network model verification and enhancement

Crowdsourcing is typically used to perform small, highly constrained tasks. In contrast, the sbv IMPROVER project aims at a higher-level task: verifying methods and data used in systems biology using a crowdsourcing approach. Contributions from the crowd on specific topics such as computational methods for gene signature extraction (24) or investigation of the concept of species translatability (25) are encouraged by the design of challenges (26) in a similar fashion to DREAM challenges (http://dreamchallenges.org/). To explore crowdsourcing beyond computational tasks, sbv IMPROVER launched the Network Verification Challenge (NVC) to leverage crowdsourcing for the verification and enhancement a set of biological network models. The substrate of the verification was a set of 50 network models describing important pathways in lung biology ranging in size between 30 and 400 nodes. The set of networks offers a framework for understanding disease and describes the relationships between molecular mechanisms involved in the regulation of biological processes (27).What makes this set of networks different from others such as KEGG (28) or Wikipathways (29) is that clear network boundaries are set prior to building the networks, in terms of pathways and context (e.g. healthy versus disease or liver versus lung). The building of the networks was as follows: published literature was manually curated to extract causal relationships between entities described in the Biological Expression Language (BEL) and then datasets were used to enhance the networks. BEL allows the representation of precise biological relationships in a computable and standardized format. Importantly, in addition to the entities and their relationship, BEL makes it possible to capture as evidence text the publication and the context in which the relationship was demonstrated, which is essential to ensure that the networks are complying with the predefined biological boundaries. Since each causal edge needs to be supported by at least one piece of evidence, the building of networks with hundreds of edges requires substantial curation. The initial network building was supported by a large proprietary knowledgebase (Selventa, USA), which was complemented by additional manual curation of evidence when needed. Nowadays, extraction of causal relationships from publications can be done to a large extent in semi-automated fashion with text mining tools such as BELIEF, which detects and tags entities and proposes triplet relationships to the curator (30). The part that remains very challenging for text mining methods is the automatic extraction of context information, as this information may be found far from the sentence/figure that demonstrates the relationship itself.

**Figure 5.**
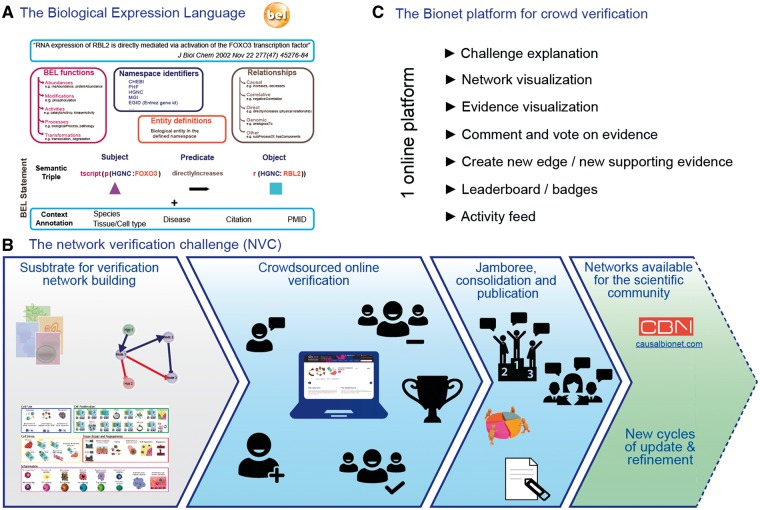
sbv IMPROVER framework.

In order to ensure a comprehensive and up-to-date set of biological network models that cover a wide range of biological signaling, crowdsourcing was used to (i) gather input from the scientific community related to the relevance of the evidence already present in the network and (ii) add new nodes and edges to the networks (Figure 5). Crowdsourcing proved to be a powerful tool for efficiently gathering feedback from a wide audience with expertise in many biological areas. The Network Verification Challenge was open to all members of the scientific community to check and verify the evidence supporting the edges of these networks. A reputation-based system and a leaderboard were used to encourage participation and to highlight the most relevant contributions. Participants were encouraged to make many contributions, but the validity of these contributions, as judged by the peers on the platform, was the key to getting a high ranking in the leaderboard. After months of on-line verification, some of the evidence-based contributions were accepted, while others were rejected. Controversial edges, i.e. the ones for which the crowd did not reach a consensus regarding their validity, were discussed in a face-to-face jamboree where the top 20 participants in the leaderboard were invited. This invitation was certainly a key incentive for scientists to contribute. This event allowed participants to consolidate their votes and discuss additional changes. Finally, as an additional incentive much appreciated by the participants, the same top 20 participants were invited to share authorship of a publication that describes the verification and the set of curated networks (27). Importantly, the refined network model set was made available to the scientific community through the causalbionet database (http://causalbionet.com), so that the activity of the scientists who participated in the challenge benefits them and their peers.

The network verification challenge demonstrated the usefulness of crowdsourcing for biological network refinement and verification. It likely would not be, in its current form, a viable option for new network curation, and other initiatives, such as WikiPathways are certainly more appropriate for engaging a larger crowd for this particular task (29). The biggest difficulties for participants were learning BEL and understanding the network boundaries. Interestingly, a survey of the participants showed that they were mainly motivated by the invitation to the jamboree and the opportunity to coauthor a publication. Moreover, many graduate students were motivated by the chance to learn about network biology as a tool and the biology described in the networks. The challenge mostly attracted biologists who are used to reading articles to extract mechanistic information. Importantly, the set of evidences curated in the context of these networks was used as the gold standard to score text mining tools in the track 4 of Biocreative V (31). It would be very interesting to see the difference that the involvement of professional curators could bring. An additional avenue that may be of interest for this verification platform in the future would be to create an interface that would allow a direct feed of contributions from text mining platforms.

## Crowdsourcing and curation of metadata for metagenomics

Metagenomics is the study of genetic material extracted directly from microbial communities in environmental samples. It enables the survey of the different microorganisms present in any environment (e.g. aquatic, soil, etc.), and addresses two main questions: who is there and what are they doing? Since metagenomics bypasses the need for culturing, it allows tapping into the uncultured diversity of microbial life.

The recent advances in sequencing technology have led to an unprecedented increase of sequencing projects, both for isolate organisms and environmental communities (metagenomics). The Genomes OnLine Database (GOLD) (32) is a comprehensive resource for information on genome and metagenome sequencing projects and their associated metadata. In order to meet the increasing complexity of projects, they have been reorganized into four different levels: studies, biosamples, sequencing projects and analysis projects. The study represents the overall umbrella under which one or several sequencing projects may be performed. The biosample represents the physical entity, the material taken from the environment. From a single biosample, we may be able to extract DNA for a metagenome, extract RNA for a metatranscriptome, isolate a number of organisms or identify a number of single cells. For each of the sequencing projects, we could run a number of different analysis projects, for example multiple different assemblies or annotations, or merging different sequencing projects into a single combined assembly. This multi-level organization of the project information has several advantages for data provenance and reporting. It enables connecting multiple sequencing projects to the same biosample, tracking multiple analyses to the same sequencing project and processing multiple analysis types from multiple sequencing projects.

Currently, GOLD has over 25 000 studies, 98 000 biosamples, 92 000 sequencing projects and 90 000 analysis projects. Each project level has a large number of associated metadata, some of which apply only to isolate genomes, some only to metagenomes and a large number to both.

By definition, metagenomes have a large number of organisms, the majority of which are usually uncultured and unknown. As a result, contextual information of the environment from which the biosample was taken (such as location, habitat and physiological properties) is extremely important and analogous to the importance that taxonomy has for the isolate organisms.

In this respect, metagenomics can be viewed as the interface of organisms/populations and the environment. The integration of the sequence data (i.e. metagenomes) with the contextual data (i.e. environmental metadata), is key for the interpretation and comparative analysis of metagenomes. Accordingly, paraphrasing Dobzhansky’s famous quote for evolution, we can say that nothing in metagenomics make sense except in the light of metadata.

For isolate organisms, particularly for the type strains of microbes, there are usually sufficiently rich metadata available in the literature, or in the databases of the Global Bioresource Centers (culture collection centers). In this case, crowdsourcing could significantly contribute to the process of metadata curation, provided that there are specific metadata standards for capturing specific sets of metadata fields from the literature to databases. For non-type strains and for metagenomes, the situation is significantly harder, due to the lack of sufficient documentation and associated descriptions in the publications. For example, often, genome sequences of non-type strains are either not published, or if published, there is very limited information on the isolation source or other physiological properties of the organisms or the environment from which they were isolated. Similarly, a large number of metagenomic studies are either not published, or the publication often has limited information on the environment from which the sample was taken. Crowdsourcing could again be very useful in the cases where there is some information available in the publication.

Overall, the most important information for a metagenome, and one that would enable the grouping and comparison with all other metagenomes, even in the absence of all other metadata, is the ecosystem from which the biosample was isolated. Accordingly, GOLD has developed a taxonomy-like system for metagenomics, based on the habitat classification.

The difficulty of obtaining adequate (computable) metadata for genomic sequences, especially related to environmental samples, requires special mention. There are two potential obstacles: finding the metadata, especially if it is not entered at the time that sequence data is deposited; in this case, it has to be retrieved from published articles or from a free text descriptive field. The second obstacle is to put the data into some kind of computable form using a controlled vocabulary or ontology. Better documentation and support during the submission process would make the process easier for the data submitters/authors.

Capture of metadata is a critical, community-wide problem. Without the contextual information, full interpretation and reuse of data sets becomes impossible. Recently there have been significant advances in tools to extract taxonomic and environmental data from free text (33, 34). In light of these advances, capture of metadata might be amenable to curation by a combined text-mining/crowdsourcing approach as discussed in earlier, or possibly even by gamification. Publishers could play a role, by checking that appropriate data and metadata have been deposited prior to acceptance of an article, but this imposes a significant extra burden on publishers. Given the NIH mandates that allow publications to follow years after the sequences have been submitted, it is difficult to do retrospective metadata capture, although crowdsourcing might enable some of this retrospective annotation.

Another possibility would be for the database owner not to release an ID until both data and metadata have been deposited. This imposes a significant burden on those responsible for maintaining repositories and there is tension between encouraging deposition of minimal data and waiting to get more complete data and metadata.

One approach might be to create a crowdsourcing ‘leaderboard’ approach (a la sbv Improver challenge) that would rate people/researchers and reward them when proper metadata is added. Alternatively, a game could be created to encourage people to add metadata for each article that they read, perhaps with small rewards or recognition (e.g. mentioning name of contributors), to encourage collaboration among researchers, authors, publishers and data depositors.

## Challenges and next steps

This paper discusses crowdsourcing at the intersection of two fields (natural language processing/text mining and biocuration). Overall, the consensus was that crowdsourcing can be a useful tool in curation of both types of data—linguistic and biological—provided that the task is appropriately scoped, the participants have or can gain the appropriate level of expertise and are motivated to do a good job through appropriate rewards. Reward mechanisms include contributing to citizen science, building reputation or monetary rewards, or even simply having fun, depending on the task and crowdsourcing approach.

There are different kinds of crowdsourcing, involving different participants, tasks of different complexity and with different applications. For community editing applications (as exemplified by the sbv Improver case study), the approach is to enable participants with significant prior background to review candidate biological relations; the participants can be motivated with a game-like competition and rewarded by outcomes such as reputation building, participation in a conference and collective authorship of publications. Participants may also be recruited at large, with the application itself providing some training and feedback, as for ZombiLingo, where the participant learns a new skill and/or has fun, while the developer of the application can gain large amounts of training data to create better models. Another approach is paid micro-task work, where participants can be easily recruited via a crowd labor platform, such as Amazon Mechanical Turk, with minimal requirements for task-specific expertise. This shows promise for rapid cost-effective collection of data; the main challenges are achieving quality adequate to the intended purpose, scoping the micro-tasks appropriately and minimizing set up costs. One limitation of micro-tasking is the need to present manageable small tasks that are visually appealing and can be answered within a short period of time. A well-designed interface can train a task contributor to become a task expert, even when the person is not a domain expert. Recent experiments show that good instructions can be very short, and yet still yield good performance, even on a conceptually complicated task. The results of the sbv IMPROVER project suggest that with this approach, quite high-level goals can be reached.

Some areas, such as metagenomics, have glaring gaps in metadata—these would be particularly good application areas for crowdsourcing, provided that the necessary information can be exposed to participants via extraction from journal articles and/or project descriptions. Alternatively, it may be possible to apply the kinds of interfaces developed for micro-tasking to elicit metadata from contributors at the time of data deposit.

A major challenge for crowdsourcing is to maintain quality; user input is useful only if it can be periodically validated, whether against a gold standard, rules of the game or other users’ input. In particular, the use of aggregated crowd judgments to validate input is a key strength of crowdsourcing, and creates an important training opportunity. The various approaches (including gaming, community editing and micro-tasking) have built in feedback/assessment mechanisms as part of the quality control process that could enable participants to receive feedback and improve their own skills over time, as well as contributing better data. Through these mechanisms, crowdsourcing can become both an educational/training tool and even a recruiting tool, where the best contributors are engaged on a more regular basis to perform tasks such as curation.

## Disclaimer

This report was prepared as an account of work sponsored by an agency of the United States Government. Neither the United States Government nor any agency thereof, nor any of their employees, makes any warranty, express of implied, or assumes any legal liability or responsibility for the accuracy, completeness, or usefulness of any information, apparatus, product, or process disclosed, or represents that its use would not infringe privately owned rights. Reference herein to any specific commercial product, process, or service by trade name, trademark, manufacturer, or otherwise does not necessarily constitute or imply its endorsement, recommendation, or favoring by the United States Government or any agency thereof. The views and opinions of authors expressed herein do not necessarily state or reflect those of the United States Government or any agency thereof. 

## Funding

BioCreative has been partially funded by NIH/NIGMS R13-GM109648-01A1, by the National Institutes of Health Intramural Research Program, National Library of Medicine and by DOE grant DE-SC0010838. ZombiLingo is funded by Inria and by the French Ministry of Culture through a DGLFLF grant to KF. sbv IMPROVER is funded by Philip Morris International. KBC's work was supported by grants NIH 2R01 LM008111-09A1 NIH 2R01 and LM009254-09 NIH to Lawrence E. Hunter, 1R01MH096906-01A1 to Tal Yarkoni, and NSF IIS-1207592 to Lawrence E. Hunter and Barbara Grimpe. Funding for open access charge: The MITRE Corporation. 

*Conflict of* *interests*: The authors declare that they have no competing interests. Stéphanie Boué is an employee of Philip Morris International.
